# Fine-mapping of angular leaf spot resistance gene *Phg*-*2* in common bean and development of molecular breeding tools

**DOI:** 10.1007/s00122-019-03334-z

**Published:** 2019-04-11

**Authors:** Juanita Gil, Diana Solarte, Juan David Lobaton, Victor Mayor, Santos Barrera, Carlos Jara, Steve Beebe, Bodo Raatz

**Affiliations:** 1 Universidad de Los Andes, Bogotá, Colombia; 2 Universidad del Tolima, Ibagué, Colombia; 3 Environmental and Rural Sciences School, University of New England, Armidale, Australia; 4 Progeny Breeding, Madrid, Colombia; 5 Centro Internacional de Agricultura Tropical (CIAT), Bean Program, Palmira, Colombia

## Abstract

**Key message:**

The Common Bean Angular Leaf Spot Resistance Gene *Phg*-*2 was* fine-mapped to a 409-Kbp region, and molecular markers for breeders were developed and validated in field experiments.

**Abstract:**

Common bean (*Phaseolus vulgaris* L.) is an important food legume in Latin America, Asia and Africa. It is an important source of protein, carbohydrates and micro-minerals, particularly for smallholder farmers. Common bean productivity is affected by angular leaf spot (ALS) disease caused by the pathogenic fungus *Pseudocercospora griseola*, resulting in significant yield losses, particularly in low-input smallholder farming systems in the tropics. The ALS resistance gene *Phg*-*2,* which was found in several highly resistant common bean genotypes, was investigated in crosses between Mesoamerican pre-breeding lines and elite Andean breeding lines. Next-generation sequencing (NGS) data sets were used to design new SNP-based molecular markers. The *Phg*-*2* locus was confirmed to be the major locus providing ALS resistance in these crosses. The locus was fine-mapped to a 409-Kbp region on chromosome 8. Two clusters of highly related LRR genes were identified in this region, which are the best candidate genes for *Phg*-*2*. Molecular markers were identified that are closely linked to the *Phg*-*2* resistance gene and also highly specific to the donor germplasm. Marker-assisted selection (MAS) was used to introgress the *Phg*-*2* resistance locus into Andean breeding germplasm using MAB lines. The usefulness of molecular markers in MAS was confirmed in several field evaluations in complex breeding crosses, under inoculation with different ALS pathotypes. This project demonstrates that NGS data are a powerful tool for the characterization of genetic loci and can be applied in the development of breeding tools.

**Electronic supplementary material:**

The online version of this article (10.1007/s00122-019-03334-z) contains supplementary material, which is available to authorized users.

## Introduction

Common bean (*Phaseolus vulgaris* L.) is the most important food legume for direct human consumption around the world (Broughton et al. 2003). Common bean is grown in the tropics in Eastern/Southern Africa as well as Latin America and Asia. In Africa and the Americas alone, production exceeds 13.5 million t/year (FAO 2016). Beans are of particular importance to smallholder farmers that depend on the staple for its calories, protein and nutritional value.

Bean production is affected by many constraints including pests and diseases as well as abiotic stresses such as drought or low soil fertility. Smallholder farming systems with low inputs of agrochemicals are particularly vulnerable to these stresses. Angular leaf spot (ALS), caused by *Pseudocercospora griseola* (Sacc.) Crous and Braun [previously known as *Phaeoisariopsis griseola* (Sacc.) Ferrari (Crous et al. 2006)], is a major production constraint of common bean in the tropics (Wortmann et al. 1998). Being a widespread and devastating disease, ALS can cause yield losses up to 80% (Schwartz et al. 1981; Correa-Victoria et al. 1989).

Common bean germplasm is divided into two gene pools that were domesticated individually, the mainly large-seeded Andean gene pool, and the small-to-medium-seeded Mesoamerican gene pool (Gepts and Bliss 1985; Bitocchi et al. 2012). Accordingly, *P. griseola* pathotypes can also be classified into two groups, namely Andean and Mesoamerican, which co-evolved with the gene pools of its host (Guzmán et al. 1995). The Andean class of isolates are mainly pathogenic on Andean beans, while the Mesoamerican isolates are more pathogenic on Mesoamerican beans but also affect Andean beans, showing a greater diversity of virulence (Pastor-Corrales et al. 1998).

Many efforts have increased the knowledge about pathotypes, have characterized resistance genes and have generated molecular markers for marker-assisted selection (MAS) (recently reviewed by Nay et al. 2019). Five major resistance genes have been confirmed, named *Phg*-*1* to *5*, identified in different resistance sources from both gene pools of common bean. *Phg*-*2* at the end of chromosome 8 was reported in several highly resistant genotypes and probably represents the strongest ALS resistance locus characterized so far providing resistance to many isolates. It was originally discovered in the Mesoamerican cultivar Mexico 54 as a single-dominant gene and named *Phg*-*2* by Sartorato et al. (1999) studying a cross between Mexico 54 × Rudá. Genetic mapping revealed that the RAPD markers OPN02^890^, OPAC14^2400^ and OPE04^650^ were linked to *Phg*-*2*, at 5.9, 6.6 and 11.8 cM, respectively (Sartorato et al. 2000). A resistance locus at the end of chromosome 8 was also found in the Mesoamerican lines Cornell 49-242, MAR 2, G10474, BAT 332 and G10909 (Ferreira et al. 2000; Nietsche et al. 2000; Caixeta et al. 2003; Mahuku et al. 2004, 2011), which may represent allelic variations of *Phg*-*2* to Mexico 54. Further SCAR markers PF5, PF9, and PF13 were developed during these efforts, which are also linked to the *Phg*-*2* gene.

Breeding for disease resistance is considered the most sustainable method to reduce production losses due to diseases. Breeding for ALS resistance has mainly relied on phenotypic selection (Arantes et al. 2010), but occasionally MAS has been employed (Namayanja et al. 2006). A variety of molecular marker systems has been used to study disease resistance in common bean. Recently, SNP markers are becoming the dominant marker type as they are the most abundant and amendable to high-throughput genotyping platforms. SNP-based markers tagging ALS resistance have been converted from previous marker systems. Markers that are tightly linked to the resistance gene and highly specific for the resistance source are available for *Phg*-*4* (Keller et al. 2015), and whole-genome sequencing data were used to develop germplasm-specific markers for five ALS resistance loci (Lobaton et al. 2018).

This study aimed to characterize and fine-map the *Phg*-*2* gene and to develop molecular breeding tools to utilize this ALS resistance gene in breeding. Mesoamerican pre-breeding lines carrying *Phg*-*2* from the resistance source G10474 were available to introgress the resistance locus into Andean elite germplasm. Genetic fine-mapping was used to delimit the *Phg*-*2* locus. Breeder-ready markers were developed and validated in complex genetic backgrounds evaluated for disease resistance in several field trials.

## Materials and methods

### Plant material

Several Mesoamerican pre-breeding lines coded MAB (after “mancha angular” the Spanish word for ALS) with the pedigree (MD 23-24 × (G4691 × G10474)) × (G4090 × 9824-56-2) were developed at the International Center for Tropical Agriculture (CIAT, Colombia). The elite Mesoamerican breeding line MD 23-24 was crossed with ALS resistance sources G10474 and G4691 and sources for Bean Common Mosaic Virus. Different resistance sources were used in the crosses, following a strategy to pyramid several resistance genes in the same germplasm. The MAB lines selected for this study were: MAB 348, MAB 349, MAB 351, MAB 352, MAB 353, MAB 354, and MAB 484, due to their observed resistance to the disease in the field (CIAT location Santander de Quilichao, Colombia). Only the line MAB 484 has a different pedigree ((MAB 163 × SER 31) × (SEN 22 × SER 7)) compared to the other MAB lines described above.

To further introgress ALS resistance into beans with Andean seed type, MAB lines were crossed to CAL 96 and other elite Andean breeding germplasm. The populations used for fine-mapping in this project are crosses between the seven MAB lines and CAL 96 (pedigree: MAB × CAL 96). CAL 96 is a commercial variety from the Andean gene pool valued for its high-quality grain and its wide adaptation in East and Southern Africa, which is susceptible to ALS. As generations were advanced, plants that were heterozygous for associated markers were identified to create residual heterozygous lines (RHL), as a tool for fine-mapping.

### ALS phenotyping

Phenotypic evaluations under greenhouse conditions were carried out at CIAT headquarters in Palmira, Colombia (latitude 3° 29′ N; longitude 76° 21′ W; 965 masl, average temperature 23 °C).

Four different isolates of the pathogen *P. griseola* were tested to evaluate the response of the samples to the infection. Colombian isolate Pg331-1 from Santander de Quilichao belonging to race 63–63 was selected for the screening of the population because the disease response showed clear differentiation between resistant and susceptible plants. For field inoculations, isolates are used that have been collected at those same locations.

For phenotypic evaluations during the fine-mapping process, plants were grown in the greenhouse and inoculated with the isolate 17 days after sowing following CIAT’s standard evaluation procedures (Castellanos et al. 2011). Disease development was recorded 10, 12, 14 and 17 days after inoculation using a standard visual scale where scores 1–3 represent resistant, 4–6 intermediate and 7–9 susceptible plants (van Schoonhoven and Pastor-Corrales 1987). The area under disease progress curve (AUDPC) was calculated and used to classify plants into resistant (AUDPC ≤ 20) or susceptible (AUDPC > 20) (Mahuku et al. 2004).

ALS was evaluated in the field in Darien, Colombia (3° 55′N latitude, 76° 28′W longitude, 1457 masl), and Santander de Quilichao (3° 06′N latitude, 76° 31′W longitude, 990 masl) applying three ALS inoculations.

In Darien, F3:4 populations were sown in June 2014 in two non-replicated experiments. The first experiment was inoculated with a mix of Mesoamerican ALS isolates Pg348-1 (race 31–47), Pg353-1 (63–47), and Pg355-1 (15–47), and the second experiment was inoculated with a mix of Andean ALS isolates Pg401-1 (15–0), Pg420-1 (63–0), and Pg422-1 (30–0). ALS lesions on leaves were evaluated 47, 61 and 75 days after sowing, phenotypically segregating lines were ignored, mean values of three evaluations were analyzed. ALS was scored in pods once 89 days after sowing.

In Quilichao, Mesoamerican F1:2 populations were sown in March 2017, without replications. Two rows of 3 m (3.60 m^2^) of each material were sown. Inoculations were carried out 24, 31 and 38 days after sowing with a mix of isolates Pg3 (race 63–0), Pg15 (7–35), Pg32 (31–55), Pg61 (13–63), Pg318 (15–39), Pg254 (31–47), and Pg66 (63–0).

ALS lesions on leaves were evaluated 57 days after sowing; for segregating rows, the highest scoring plant was recorded. Individual selections of these lines were sown for evaluation in the following generation in Darien in October 2017, in a non-replicated trial in rows of 2.80 m. ALS lesions on leaves were evaluated 55 days after sowing; segregating rows were ignored in the analysis.

### Read alignment, variant detection, genotyping and merging of variants

Genotyping-by-sequencing (GBS) and whole-genome sequencing (WGS) paired-end raw reads were demultiplexed using the Demultiplex module of NGSEP (Duitama et al. 2014). Every read was scanned for the first 6-mer of the adapter sequence, and reads were then trimmed to only retain the common bean sequence. Trimmed reads were aligned to the *Phaseolus vulgaris* v2.1 reference genome (https://phytozome.jgi.doe.gov/pz/portal.html, accessed 2017) (Schmutz et al. 2014), using bowtie2 with default parameters (Langmead and Salzberg 2013). Alignments were coordinate-sorted and indexed using SAM Tools (Li et al. 2009). Variant discovery was performed by running the NGSEP FindVariants module. The maximum base-quality score was set to 30, and the minimum quality for reporting a variant was set to 40; five bases at the 5′ end and five bases at the 3′ end of each read were ignored. Distinct to WGS, as required for GBS data, the detection of repetitive regions, copy number variants (CNVs) and other structural variants was turned off. Correspondingly, due to the nature of GBS experiments, the maximum number of reads allowed to start at the identical position which was raised to 100. The prior heterozygosity rate was set to 10^−4^, and minor allele frequency was set to 0.05 as in other *Phaseolus* populations (Perea et al. 2016; Lobaton et al. 2018). In order to obtain the list of variant sites from GBS populations in combination with the WGS sequencing data, the variant (VCF) files developed for WGS and GBS data sets were combined using the MergeVariants module. Afterward, all samples were genotyped at the variant sites by running the FindVariants module again, keeping all parameters unchanged except for the minimum variant quality score, which we set to 40 to retain higher-quality genotype calls. Finally, the MergeVCF module was used to join all the VCFs into a single file gathering the genotyping information of the whole experimental population.

### Design of molecular markers

SNP-based molecular markers were used to fine-map the *Phg*-*2* gene locus. Based on the physical position of SCAR marker PF5 (Mahuku et al. 2004), SNPs were initially selected covering a region of 1.74 Mbp, which represents ~5 cM up- and downstream of the reported position for PF5. Further markers were developed at different stages of the fine-mapping process, based on improved information on the *Phg*-*2* location.

Taking advantage of several sets of next-generation sequencing (NGS) data, SNPs were either selected from a panel of common bean genotypes sequenced by WGS (Lobaton et al. 2018), or from a panel of 600 breeding lines genotyped by GBS using the ApeKI enzyme (Elshire et al. 2011). The genotyped panel included three of the parental MAB lines MAB 348, MAB 349 and MAB 484, as well as G10474 and CAL 96.

For breeding marker development, SNPs were sought that were unique for the MAB lines and/or G10474 in a panel of Mesoamerican and Andean genotypes. To develop markers for the fine-mapping study, the extensive list of SNPs was utilized that differentiates Mesoamerican from Andean gene pools (Lobaton et al. 2018), which contains SNPs that are polymorphic in most Mesoamerican × Andean mapping populations. The SNP marker ALS_08_62193174 (or sc267437, polymorphism also referred to as sc00091ln623366_267437_T_G_85408661 or ss715646759) was developed based on a SNP available from the BARCBean6K_3 SNP chip (Cichy et al. 2015; Song et al. 2015). All SNP-based markers used are listed in Online Resource 2.

A combined panel of 600 lines genotyped by WGS and GBS was utilized to select further candidate SNPs for breeding markers. MAB 348, MAB 349 and G10474 were defined as resistance source lines, and 22 lines that had previously been reported to show ALS resistance were set aside as extended positive controls (Online Resource 5), Andean resistance sources G5686 and AND 277 were ignored, because Andean germplasm may have other resistance genes and thereby confuse the association. The remaining lines were used as the susceptible set. Polymorphisms in the region between 61,879,947 and 62,139,256 bp on chromosome 8 were filtered, selecting SNPs (1) with homozygous genotype calls for the source lines,( 2) the same genotype call for all source samples, (3) that were biallelic, and (4) with a source allele frequency in the rest of the panel below 10%.

### DNA extraction and genotyping

Total genomic DNA from all the plants sampled in the greenhouse was extracted either from young trifoliate leaves or from seed powder in 96-well plates using liquid nitrogen and an SDS/NaCl-buffer-based DNA extraction Mini-Prep protocol (modified from King et al. 2014). DNA quality and concentration were visually estimated by electrophoresis on 1% agarose gels. DNA was then diluted 1:10 with distilled water for PCR.

For the field experiments, DNA was extracted from young tissue in 96-well plates. One disk from young trifoliate leaves from each plant was obtained using a hollow punch and then placed into a well of the plate and stored at − 80 °C. DNA was extracted with the alkaline protocol, 100 µl extraction buffer (50 mM NaOH, 1% Tween 20) was added to each well containing the frozen tissue and the plate was placed in a water bath with boiling water for 10 min. Then, 50 µl neutralization buffer (100 mM Tris HCl, 1.7 mM EDTA, pH 7.3) was added to each well and the plate mixed by vortexing at moderate speed. DNA was diluted 1:10 with distilled water for PCR.

### SCAR genotyping

The SCAR marker PF5 was amplified by PCR in 15 μl reaction volumes containing 5 µl of genomic DNA, 1X PCR Buffer [10 mM Tris–HCl pH 8.8, 50 mM KCl, 0.8% (v/v) Nonidet P40 (Fermentas)], 2.5 mM of MgCl_2_ (Fermentas), 0.4 mM of dNTPs mix (Promega), 0.2 μM of each primer (forward and reverse), and 0.15 µl of Taq polymerase (laboratory made). The PCR was performed on an Eppendorf Pro Mastercycler under following conditions: a first denaturation step at 94 °C for 5 min, followed by 35 cycles of denaturation at 94 °C for 30 s, annealing at 60 °C for 30 s, and extension at 72 °C for 45 s, and then a final extension step at 72 °C for 10 min before cooling down to 4 °C. PCR products were visualized on 1.5% agarose gels in 1X BS buffer on a HORIZON 20·25 gel electrophoresis system. GelRed™ (Biotium Inc., Hayward, CA, USA) was added to the gel for visualization of the DNA bands on an ultraviolet transilluminator (Foto/UV26, Fotodyne Incorporated, New Berlin, WI, USA).

### SNP genotyping by melting temperature Tm-shift (Tms)

Primers were designed according to Wang et al. (2005) using the software Primer 3 (Untergasser et al. 2012). Amplification and melting point analysis for allele determination were performed on a fluorescence-detecting thermocycler (CFX384 Real-Time System, Bio-Rad) with EvaGreen fluorescent dye (Biotium, Fremont, CA, USA). The PCR volume was 15 µl containing 5 µl of genomic DNA, 1X PCR Buffer, 2 mM of MgCl_2_, 0.2 mM of dNTP mix, 0.2 μM of each primer (two allele-specific forward primers and the common reverse primer), 0.8X EvaGreen, and 0.1 µl of Taq polymerase (laboratory made) under the following thermal profile: a first denaturation step at 94 °C for 3 min, then 35 cycles of denaturation at 92 °C for 20 s, annealing for 20 s (temperature specific to each primer trio), extension at 72 °C for 20 s, and finally, 1 min at 95 °C. The melting curve step ramping from 70 to 95 °C and increments of 0.5 °C/20 s under fluorescence detection followed the amplification.

### SNP genotyping by high-resolution melting (HRM) analysis

HRM primers were designed flanking a SNP with an amplicon size not exceeding 100 bp. PCR amplification was carried out as described before for Tm-shift genotyping on the CFX384 Real-Time System with the difference that the amplification product was melted ramping from 65 to 95 °C and incrementing 0.2 °C/20 s. HRM analysis was performed using the software Bio-Rad Precision Melt Analysis 1.2 (Bio-Rad).

### SNP genotyping using LGC KASP markers

KASP™ chemistry is based on using three primers, two allele-specific and a common reverse primer, similar to Tm-shift, whereas different tags are used to incorporate fluorescence-labeled oligos. KASP assays were purchased from the commercial provider LGC genomics (Hertfordshire, UK) (http://www.lgcgroup.com/products/kasp-genotyping-chemistry) and run on the CFX384 Real-Time System following the provider’s instructions for 5 µl reaction volumes.

### Identification of candidate genes

Annotations of genes in the fine-mapped region on chromosome 8 were downloaded from the phytozome database (https://phytozome.jgi.doe.gov/pz/portal.html, accessed 2017). Annotations were evaluated for their reported involvement in disease resistance, and such genes were identified as candidates.

## Results

### *Phg*-*2* segregates as a single gene in crosses with MAB lines

The MAB lines are pre-breeding lines that were selected for bush growth habit, small red seed and good agronomic performance, combined with repeated phenotypic ALS resistance under field conditions. Most MAB lines were found to be resistant to several ALS strains (Table 1).

**Table 1:** Phenotypic ALS evaluations and genotyping data of MAB lines and other parental germplasm

Line	Pg3^a^	Pg331-1†	Pg335-1†	Pg347-1†	Phenotype	PF5 (pos. 61,251,609 bp)	ALS_08_61658896	ALS_08_61705350	ALS_08_61730261	ALS_08_61731208	ALS_08_61825915	ALS_08_61901622	ALS_08_62047925	ALS_08_62139256	ALS_08_62170288	ALS_08_62193174
MAB 348	1.0	2.7	2.9	2.2	R	+	C	T	A	G	A	T	C	A	G	G
MAB 349	1.1	2.1	3.7	2.2	R	+	C	T	A	G	A	T	C	A	G	G
MA B 351	1.2	1.8	2.2	1.6	R	+	C	T	A	G	A	T	C	A	G	G
MAB 352	1.2	1.1	2.3	2.9	R	+	C	T	A	G	A	T	C	A	G	G
MAB 353	1.2	1.9	3.4	2.3	R	+	C	T	A	G	A	T	C	A	G	G
MAB 354	1.0	2.4	2.7	3.4	R	+	C	T	A	G	A	T	C	A	G	G
MAB 484	1.3	2.3	8.0	8.6	Isolate dependent	+	C	T	A	G	A	T	C	A	G	G
G10474	1.0	1.0	1.0	1.0	R	+	C	T	A	G	G	T	C	A	G	G
G4691	1.2	5.2	7.3	2.7	Isolate dependent	+	C	T	NA	G	G	G	C	G	T	G
MD 23-24	1.0	5.1	7.3	2.5	Isolate dependent	+	C	T	A	G	A	G	C	G	G	T
G4090	1.0	8.0	4.7	9.0	Isolate dependent	+	C	T	NA	G	G	G	C	G	G	T
9824-56-2	–	–	–	–	No data	+	T	T	G	G	G	G	C	G	T	T
CAL 96	6.2	6.7	8.5	8.4	S	−	T	C	G	C	G	G	T	G	T	T

*R* resistant, *S* susceptible, *NA* no amplification

†Mesoamerican isolates

^a^Andean isolate

To introgress ALS resistance into beans with Andean seed type, MAB lines were crossed to CAL 96 and other Andean elite lines. The inheritance of ALS resistance was characterized in these crosses evaluating seven MAB × CAL 96 cross populations in F3 generation (Table 2). A Chi-squared test was conducted to investigate whether the ratio of resistant and susceptible individuals deviates from the 5:3 ratio expected for a single-dominant resistance locus. In five populations, the observed ratio was not significantly different to the expected 5:3 ratio, in line with a single-dominant resistance gene. Two populations deviated significantly (p < 0.05) from the expected ratio, which may indicate involvement of other loci or insufficient sample size. In all cases, ALS resistance was transferred from the MAB sources to the new populations.

**Table 2:** Segregation ratios of ALS resistance in seven F3 populations of CAL 96 × MAB crosses

Cross	# Plants per population	Expected ratios resistant/susceptible (5:3)	Observed ratios resistant/susceptible	*X* ^2^	*p* value
CAL 96 × MAB 348	38	24:14	30:8	4.3860	0.036
CAL 96 × MAB 349	35	22:13	22:13	0.0019	0.965
CAL 96 × MAB 354	15	9:6	13:2	3.7378	0.053
MAB 352 × CAL 96	27	17:10	12:15	3.7556	0.053
MAB 351 × CAL 96	19	12:7	14:5	1.0140	0.314
MAB 353 × CAL 96	11	7:4	5:6	1.3636	0.243
CAL 96 × MAB 484	34	21:13	27:7	4.1490	0.042
All crosses	179	112:67	123:56	2.9501	0.086

Plants were inoculated with ALS isolate Pg331-1. Single-dominant gene hypothesis was evaluated using the Chi-squared test (*X*^2^)

**Fig. 1:**
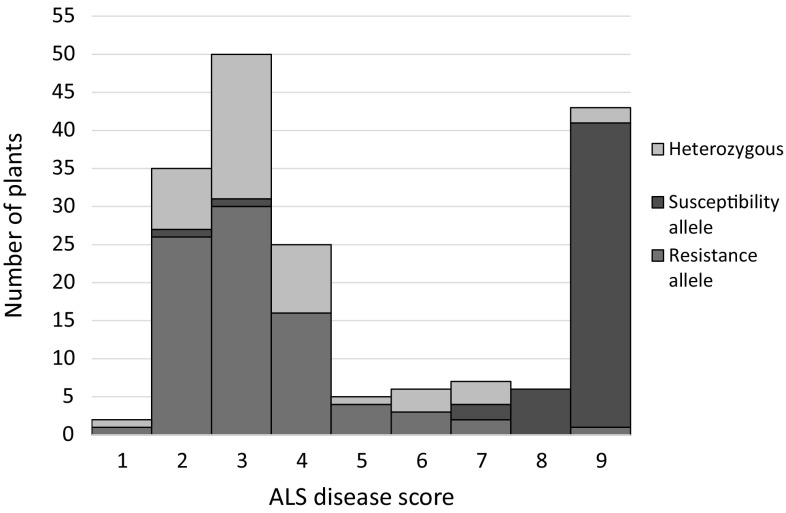
ALS disease severity scores in 179 F3 plants from seven MAB × CAL 96 crosses (Table 2), evaluated with ALS isolate Pg331-1. Color code indicates genotype at *Phg*-*2* locus evaluated with *Phg*-*2* associated marker ALS_08_62193174 (color figure online)

Co-segregation of ALS resistance with the *Phg*-*2* locus was evaluated in these 179 F3 plants (50 F2-derived families). Crosses were evaluated phenotypically and genotypically with marker ALS_08_62193174 (Fig. 1). Disease scores largely fell into two distinct classes of resistant and susceptible lines suggesting single-gene resistance. The genotype of the *Phg*-*2* marker clearly co-segregated with the phenotype, while the few non-concordant samples may be phenotypic escapes or due to recombinations. Data demonstrate that the *Phg*-*2* gene confers ALS resistance in this population.

To investigate the source ALS resistance, the parental lines of the crosses were evaluated phenotypically and genotyped with the SSR marker PF5, previously published to be linked with *Phg*-*2* (Mahuku et al. 2004), and 10 additional SNP-based markers in the vicinity (Table 1). CAL 96 is susceptible to all *P. griseola* strains; other lines show resistant disease scores (1–3) to some or all of the isolates of the pathogen. Based on the markers tested here, the *Phg*-*2* locus in all MAB shows the same haplotype as G10474 and not that of the other resistance source G4691 employed in the crosses.

### *Phg*-*2* was fine-mapped to a 409-Kbp region on chromosome 8

**Fig. 2:**
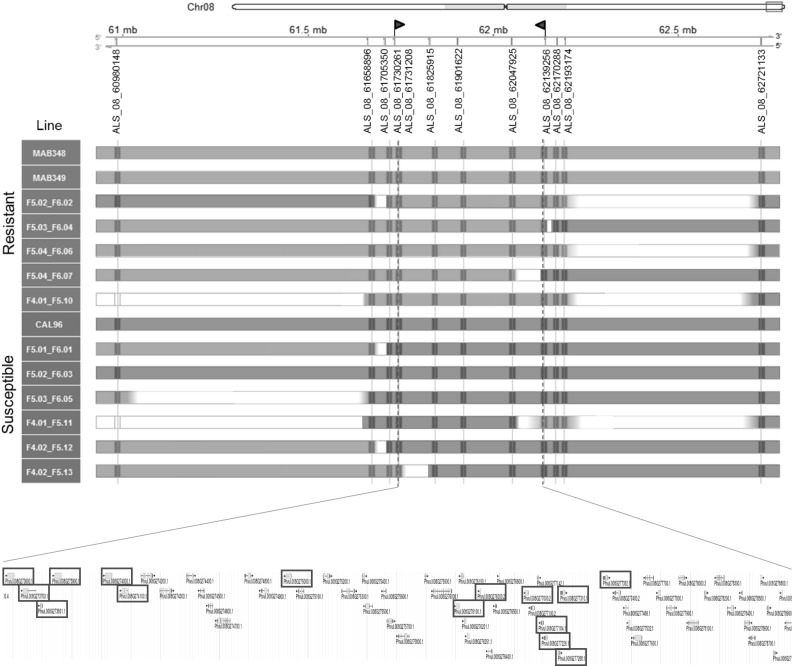
Fine-mapping of *Phg*-*2* resistance locus to a 409-Kbp region. Parents and most informative recombinants are displayed; two segregating lines from segregating families are listed where available. Line codes are composed of the plant ID that gave rise to the family (generation. plant number) followed by the ID of the phenotyped and genotyped plant. Resistant plants and resistance-associated genotypes depicted in green, susceptible in red. The fine-mapped region contains 70 genes from which 15 contain LRR motives reported to be involved in plant defense (boxed in red) (color figure online)

To fine-map the *Phg*-*2* region, we selected lines from the different MAB crosses that showed recombinations in this region for further analysis. In three of the seven MAB × CAL 96 crosses, useful recombination events were identified in the *Phg*-*2* region evaluating F4 lines (CAL 96 × MAB 484, CAL 96 × MAB 348, and CAL 96 × MAB 349). In further fine-mapping steps, only the latter two crosses were used for the analyses as descendants showed a clear phenotypic segregation and strong phenotype–genotype association for the *Phg*-*2* locus. Recombinants were identified in further generations, and if possible, several segregating descendants of each recombinant were phenotyped in the following generation, to determine whether markers on either side of the recombination were associated with the phenotype (i.e. on which side of the recombination event the resistance gene locates). Plants heterozygous at the *Phg*-*2* locus and those carrying interesting recombinations were advanced and evaluated as follows (depicted in Online Resource 1): 703 F4 seeds from 46 F3 recombinant plants were genotyped with four SNP markers covering a region of 1.6 Mbp on chromosome 8, and from those, 72 seeds were selected to be sown and evaluated phenotypically and again genotypically. Sixteen new recombination events were found and those lines were advanced to the F5 generation. Results confirmed that the *Phg*-*2* locus is flanked by markers ALS_08_60980148 and ALS_08_62721133 (Fig. 2); hence, more markers were developed in this region. Further 63 plants were evaluated phenotypically and genotypically in F5 and 320 descendant plants in F6 generation.

The *Phg*-*2* region was progressively delimited, and more SNP-based molecular markers were developed and genotyped during the process to further characterize recombinants, based on the positional information obtained in previous generations. Combining all data sets, the *Phg*-*2* locus was delimited to a 408,995-bp region flanked by markers ALS_08_61730261 and ALS_08_62139256 (Fig. 2).

### Candidate genes for ALS resistance on chromosome 8

The 409-Kbp *Phg*-*2* region identified here encompasses 70 genes (Online Resource 3). Eighteen of those are annotated as members of gene families that have been reported to be involved in plant defense; 14 are annotated to harbor LRR domains. LRR genes have been reported to directly detect and bind to pathogen proteins activating a resistance response (DeYoung and Innes 2006). Other candidate genes are annotated to encode kinases, which play a role in many signaling networks, including pathogen defense (Romeis 2001). Hence, these genes represent the likeliest candidates encoding the *Phg*-*2* gene (Table 3). The LRR genes at the start of the fine-mapped region, from Phvul.008G273600 to Phvul.008G274100, represent a cluster of closely related genes. A second group of closely related LRR genes is found between Phvul.008G276100 and Phvul.008G277352.

**Table 3:** Candidate genes for the *Phg*-*2* resistance gene

Gene ID	Start (bp)	End	Description
Phvul.008G273600^a^	61,735,204	61,737,956	Leucine-rich repeat (LRR_1)//Leucine-rich repeat N-terminal domain (LRRNT_2)//Leucine
Phvul.008G273700^a^	61,741,746	61,747,280	Leucine-rich Repeat (LRR_1)//Leucine-rich repeat (LRR_8)
Phvul.008G273801^a^	61,749,035	61,750,088	Leucine-rich repeat (LRR) protein associated with apoptosis in muscle tissue
Phvul.008G273900^a^	61,755,148	61,758,150	Leucine-rich repeat (LRR_1)//Leucine-rich repeat N-terminal domain (LRRNT_2)//Leucine-rich repeat (LRR_8)
Phvul.008G274000^a^	61,776,591	61,779,650	Leucine-rich repeat (LRR_1)//Leucine-rich repeat N-terminal domain (LRRNT_2)//Leucine-rich repeat (LRR_8)
Phvul.008G274100^a^	61,783,591	61,786,602	Leucine-rich repeat N-terminal domain (LRRNT_2)//Leucine-rich repeat (LRR_8)
Phvul.008G275000	61,853,078	61,855,595	Protein kinase domain (Pkinase)//Leucine-rich repeat (LRR_8)
Phvul.008G275100	61,858,296	61,860,853	SERINE/THREONINE-PROTEIN KINASE WNK11-RELATED
Phvul.008G276100^b^	61,925,250	61,926,777	Ras-suppressor protein (contains leucine-rich repeats)
Phvul.008G276300^b^	61,935,298	61,935,870	Leucine-rich repeat N-terminal domain (LRRNT_2)
Phvul.008G276400	61,938,810	61,940,687	Non-specific serine/threonine protein kinase/threonine-specific protein kinase
Phvul.008G276600	61,943,471	61,943,797	Non-specific serine/threonine protein kinase/Threonine-specific protein kinase
Phvul.008G277000^b^	61,955,233	61,956,464	Leucine-rich repeat (LRR_1)//Leucine-rich repeat N-terminal domain (LRRNT_2)//Leucine-rich repeat (LRR_8)
Phvul.008G277184^b^	61,961,555	61,962,863	Leucine-rich repeat (LRR_8)
Phvul.008G277226^b^	61,962,868	61,964,475	Leucine-rich repeat N-terminal domain (LRRNT_2)
Phvul.008G277268^b^	61,968,905	61,969,916	Leucine-rich repeat N-terminal domain (LRRNT_2)
Phvul.008G277310^b^	61,969,927	61,971,992	Ras-suppressor protein (contains leucine-rich repeats)
Phvul.008G277352^b^	61,987,965	61,990,190	Ras-suppressor protein (contains leucine-rich repeats)

Among the 70 genes in the fine-mapped 409-Kbp region on chromosome 8 (Online Resource 3), the 18 most likely candidate genes are shown, based on their annotation. Genes marked with ^a^ or ^b^, respectively, represent closely related gene families

Analysis of WGS data comparing G10474 with a panel of genotypes (Lobaton et al. 2018) revealed a total of 36,960 polymorphisms in the 409-Kbp region composed of SNPs, INDELs and copy number variants (CNVs). One hundred and seventeen SNPs differentiate G10474 against > 90% of the other genotypes, 101 of these SNPs are genic, 20 represent missense mutations, of which 3 appear in the likely candidates (Online Resource 4). Missense SNPs in G10474 were found in Phvul.008G275000 (positions 61,853,763 and 61,855,007 bp) and in Phvul.008G275100 (61,859,887 bp). These represent the best available candidate causal variants for the observed phenotype. However, G10474 may have further copies of this gene family, which are not present in the reference G19833 and contribute to the resistance. With the available depths and type of sequencing data, these cannot be identified.

### *Phg*-*2* markers for marker-assisted selection (MAS)

**Fig. 3:**
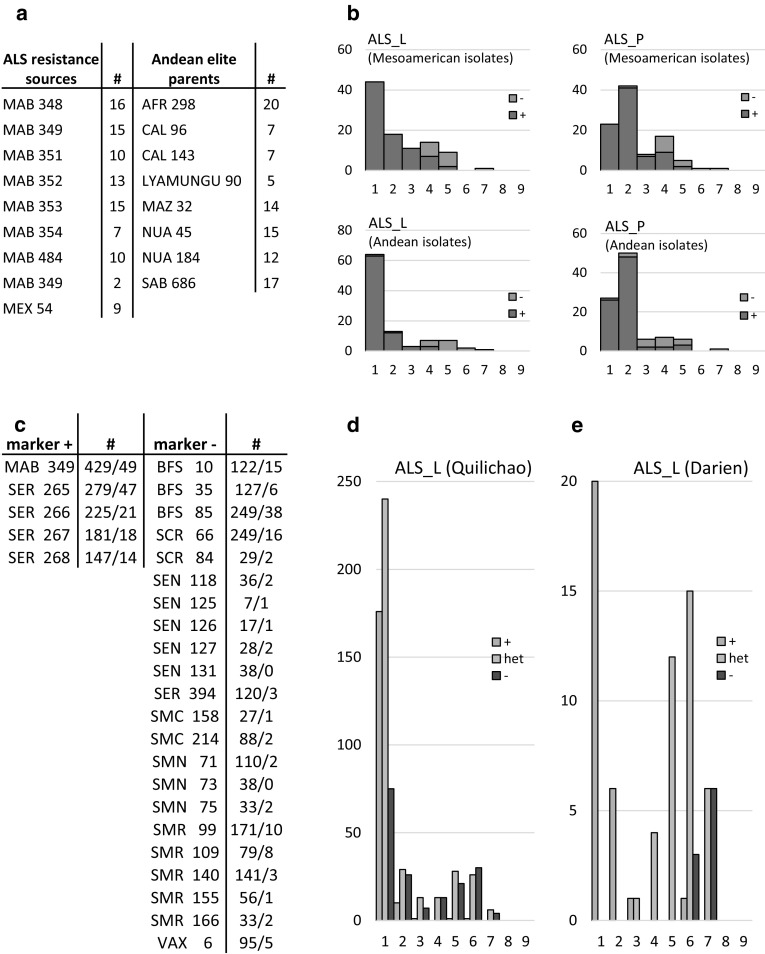
Validation of marker–trait associations in breeding lines in field experiments. **a** Parental lines of the 97 evaluated F4 breeding lines derived from bi-parental crosses between Mesoamerican ALS resistance sources and Andean elite germplasm. # indicates the number of evaluated sibling lines for which this line is a parent. **b** ALS disease scores in leaves (ALS_L) and pods (ALS_P), of 97 breeding lines inoculated with a mix of Mesoamerican or Andean ALS isolates at Darien field station. Lines that carry the ALS_08_62193174 marker resistance-associated allele are shown in green, susceptible ones in red. **c** Parental lines of 720 F2 breeding lines derived from 3-, 4-, 5-, and 6-way crosses in the Mesoamerican genepool, # indicates the number of sibling lines evaluated in Quilichao and Darien, for which this line is a parent. **d** ALS disease scores of F2 families at Quilichao field station. **e** ALS disease scores of 75 descendant F3 lines inoculated at Darien field station. Lines were genotyped in F1 generation with the ALS_08_62139256 marker, resistance-associated allele in green, heterozygous in blue, susceptible in red (color figure online)

Marker ALS_08_62193174 was used to tag the *Phg*-*2* locus for the marker-assisted introgression from Mesoamerican MAB sources into Andean breeding lines. This marker does not specifically tag *Phg*-*2* in the Mesoamerican gene pool, but it can be utilized to tag this locus in an Andean background. Ninety-seven F4 lines derived from simple crosses of 9 resistance sources with 8 elite Andean genotypes (Fig. 3a) were evaluated for ALS response in a non-replicated field trial, after inoculation with a mix of either Mesoamerican or Andean ALS isolates (Fig. 3b). Lines that carried the resistance-associated allele of ALS_08_62193174 showed higher levels of resistance in both leaves and pods for both pathotype mixes. This demonstrates the utility of the MAB resistance sources and new SNP-based markers for MAS in breeding, as the association holds up in complex genetic backgrounds and provides resistance in field evaluations with complex pathotype compositions.

Marker–trait associations were also validated in field trials with early-generation breeding lines of the Mesoamerican gene pool. Lines derived from 3-, 4-, 5-, and 6-way crosses were genotyped in the final F1 generation with the marker ALS_08_62139256. At the Quilichao field station, 720-derived F1:2 families were evaluated for ALS (Fig. 3c). Practically, all families that were genotyped homozygous for the resistance allele showed high levels of field resistance (Fig. 3d). However, many lines that carried the susceptible allele for this marker also proved to be resistant, suggesting that other alleles of *Phg*-*2* or other resistance genes also confer resistance here. Seventy-five lines from individual selections were re-evaluated in the following generation at Darien field station (Fig. 3e). Here, only lines homozygous for the resistance-associated marker allele were resistant, indicating disease pressure from pathotypes different to the Quilichao experiment; in this case, only the *Phg*-*2* allele tagged by the marker confers resistance. In summary, these results clearly confirm the marker–trait association in complex genetic backgrounds under field conditions and demonstrate its utility for MAS in breeding.

Sequencing data provide a rich polymorphism resource that can be mined for marker design. Available whole-genome re-sequencing (WGS) and genotyping-by-sequencing (GBS) data were analyzed to identify further polymorphisms for marker design with optimal positioning and differentiation between resistance sources and susceptible materials.

Polymorphisms in the region between 61,730,261 bp and 62,139,256 bp on chromosome 8 were investigated in 600 breeding lines, varieties and landraces, to identify new SNPs for markers, as described in M&M (Online Resource 5). Interestingly, sequencing data show that G10474 and MAB 348/349 seem to have a different haplotype up to about 61,879,947 bp, as several markers are observed to be polymorphic upstream of that position. GBS data are not completely conclusive, but the available data suggest that *Phg*-*2* is located in the remaining interval of 259 Kbp (61,879,947 to 62,139,256 bp).

An ideal SNP would have a high allele frequency in the group of positive controls while having a very low frequency of the resistance source allele in the remaining panel and a high number of SNP calls in the whole panel. Table 4 lists further 14 polymorphisms that differentiate resistance sources from the panel of breeding lines. These are promising to design further markers to tag the *Phg*-*2* locus in breeding crosses, in case the known markers are not suitable for certain genotyping platforms or specific genetic backgrounds.

**Table 4:** Candidate polymorphisms for ALS marker development tagging the *Phg-2* locus

Position (bp)	Ref	Alt	Positive controls	Extended positive controls		Remaining lines	
			MAB 348, MAB 349, G10474	Resistance allele frequency (%)	n	Resistance allele frequency (%)	n
61,901,182	G	T	T	4.76	21	0.00	356
61,999,071	G	A	A	5.26	19	7.64	471
61,999,210	A	G	G	5.00	20	7.08	551
62,043,108	A	T	T	75.00	16	1.65	484
62,043,144	G	A	A	75.00	16	1.83	491
62,043,203	A	C	C	83.33	18	7.17	488
62,043,204	GTTTTA	GTTA	GTTA	82.35	17	7.55	437
62,047,897	C	T	T	58.33	12	2.78	396
62,047,952	G	A	A	58.33	12	2.98	369
62,048,236	G	A	A	85.71	14	6.45	124
62,048,237	T	C	C	85.71	14	3.25	123
62,048,255	G	T	T	88.24	17	4.84	124
62,139,186	A	G	G	66.67	18	1.46	481
62,139,256	G	A	A	61.11	18	3.30	273

A panel of 600 lines with available GBS and WGS sequencing data was evaluated; results are filtered from a larger set shown in Online Resource 5

*Ref* reference allele, *alt* alternative allele, *n* indicates the number of lines in the respective group with genotype calls

## Discussion

### *Phg*-*2* was fine-mapped to a 409-Kbp region containing LRR resistance gene clusters

The *Phg*-*2* locus is the most effective ALS resistance locus reported with best cross-pathotype stability. The resistance gene *Phg*-*2* was first identified by Sartorato et al. (1999) in the resistance source Mexico 54 and subsequently discovered in several resistance sources, such as G10474 (Mahuku et al. 2004), MAR 2 (Ferreira et al. 2000), Cornell 49–242 (Nietsche et al. 2000), Ouro Negro (Queiroz et al. 2004) and G10909 (Mahuku et al. 2011). At this point, the authors assume that all of these represent the same resistance locus using the same gene name *Phg*-*2*. In case an entirely different gene confers resistance in Mexico 54 compared to G10474, e.g. a different LRR gene copy, this may have to be corrected at a later stage.

Previous studies reported the location of *Phg*-*2* at a distance of about 5 cM from marker PF5 (61,251,610 bp) (Mahuku et al. 2004), or at 3 cM distance of marker g796 (61,514,592 bp) (Miller et al. 2018). In this project, the *Phg*-*2* locus was identified much more precisely to a region of 409 Kbp. WGS data on G10474 (Lobaton et al. 2018) and lower-quality GBS data on MAB lines suggest that *Phg*-*2* is located in a 259-Kbp interval (61,879,947– 62,139,256 bp) where G10474 and the MAB lines share the same haplotype (Online Resource 5). However, GBS data should be interpreted with care and results confirmed by other genotyping methods.

Marker data show that the origin of the *Phg*-*2* locus in all MAB lines is G10474. Resistance sources G10474 and G4691 that were used in the crosses may harbor additional resistance genes, but probably these were not selected for under the applied ALS screening conditions.

Fine-mapping identified a locus with clusters of LRR genes belonging to two closely related LRR gene families. Similarly, previous resistance gene mapping efforts resulted in regions with repetitive gene clusters (Vallejos et al. 2006; Keller et al. 2015). Out of the 70 genes in the candidate region, 15 LRR genes represent the likeliest candidate genes for *Phg*-*2*. WGS data were used to identify non-synonymous SNPs in candidate genes that are associated with resistance which are candidate causal variants for the resistance. However, a further characterization of these principal candidates is challenging, as G10474 may have other or different copies of these LRR family genes. Due to the short-read lengths of the available G10474 WGS data, it is not possible to assemble such a repetitive locus, which would be required to identify the actual *Phg*-*2* gene. Long-read sequencing platforms, such as PacBio, could facilitate the assembly of such repetitive regions.

### NGS data sets are invaluable tools supporting fine-mapping strategies

The fine-mapping strategy applied in this project used residual heterozygous lines (RHLs), identifying and evaluating recombinants in consecutive generations. RHLs have been successfully employed in genetic mapping in common bean and other crops (Haley et al. 1994). This RHL method has less phenotypic noise due to the more homogeneous genetic background and is more resource efficient requiring less phenotypic evaluations. Hence, it can be applied in fine-mapping projects with lower heritability phenotypes or resource-demanding phenotyping requirements. However, this method is also more time-consuming than one-step approaches for recombinant identification, which were reported, for example, for bean common mosaic virus resistance mapping in common bean (Vallejos et al. 2006) or in soybean (Wu et al. 2018).

The growing availability of large data sets of sequencing data has a profound effect on marker design. Large databases of millions of SNPs and INDELs (Lobaton et al. 2018) contain ample polymorphisms that can be used for marker design, to achieve high marker density for nearly any genomic region. Sequencing data allowed this project to proceed with higher marker densities, where comparable previous fine-mapping effort stalled due to lack of markers (Vallejos et al. 2006; Oblessuc et al. 2013). Hence, with the availability of such data resources the success of fine-mapping is largely delimited by the genetic resolution, i.e. the size of the population and thereby the number of recombination events the can be identified and evaluated.

### Deploying *Phg*-*2* in breeding

The MAB pre-breeding lines are presented here as good sources for ALS resistance in breeding. Resistance alleles were introgressed from lines with undesirable climbing growth habits and non-commercial grain types into Mesoamerican bush breeding germplasm.

Resistance has now been transferred into a first generation of large-seeded Andean bush beans, termed AAB lines (Andean ALS-resistant bush). These lines were generated by MAS using an early marker ALS_08_62193174. Resistance was evaluated in complex crosses under field conditions in leaves and also in pods where the disease causes most economic damage in snap bean production. Pod disease scores are often not measured as screening of leaves after a few weeks is faster and easier. AAB lines are also considered pre-breeding lines as they do not yet meet the agronomic performance of elite breeding lines. *Phg*-*2* is the main resistance locus that provides resistance to different mixes of pathotypes which confirms that *Phg*-*2* is currently the most powerful ALS resistance locus.

Many past QTL studies produced markers linked to valuable loci. However, these frequently failed to be transferred to breeding applications as markers were not specific to resistance sources in other genetic backgrounds. Success as well as failure was reported by Namayanja et al. (2006). WGS and GBS data sets of large diverse panels of genotypes provide the resource to select markers of high specificity to certain resistance donor genotypes. These are then likely to tag the resistance locus not only in populations for genetic studies (usually bi-parental) but also in most other genetic backgrounds in breeding material. In this way, NGS data sets allowed this project to easily identify well positioned as well as highly source-specific markers. As the first marker ALS_08_62193174 would only effectively tag *Phg*-*2* in purely Andean backgrounds, a further marker, ALS_08_62139256, was developed and demonstrated to be associated with resistance in a complex Mesoamerican genetic background under field screening conditions. Further polymorphisms were identified in sequencing data sets (Table 4) that should allow to tag and introgress this locus into virtually any genetic background.

*Phg*-*2* is at this point the most effective ALS resistance gene available for breeding, with resistance to a broad spectrum of ALS pathotypes. However, single-gene resistance is generally not expected to provide durable resistance, as pathogen evolution is a continuous, rapid process and resistance of new varieties is often overcome rapidly (McDonald and Linde 2002). The commonly accepted concept is that resistance gene pyramids, the combination of several resistance genes into one variety, provide better long-term resistance than the most efficient single gene, as demonstrated in lentil, potato and several cereals against different pathogens (reviewed by Mundt 2018). Other ALS resistance genes are of course known, and also, G10474 may harbor further resistance genes explaining its observed broad resistance. Hence, *Phg*-*2* should be combined with other resistance genes into new varieties to ensure durable resistance.

## Conclusion

*Phg*-*2* is the most effective ALS resistance gene in common bean. The locus was fine-mapped to a 409-Kbp region on chromosome 8, where two clusters of highly related LRR genes represent the best candidate genes for *Phg*-*2*.

NGS data sets have allowed to identify SNP markers for mapping and breeding, which are well positioned as well as highly specific to the donor germplasm. Introgression of the resistance locus using MAB lines into Andean material has been demonstrated; highly specific markers are available that will most likely work in other genetic backgrounds. These tools and germplasm will aid future ALS resistance breeding activities. The NGS resources described here will be widely used to improve breeding markers for other diseases and traits. This project is an example on using NGS data as a powerful tool for the characterization of genetic loci and for their application in the development of breeding tools.

### Author contribution statement

JG is involved in fine-mapping and writing; DS performed field evaluations; JDL NGS analyzed the data; VM is involved in supervision of field evaluations and Diana’s thesis; SB performed field evaluations in Quilichao; CJ performed supervision pathology; SB contributed to the development of MAB lines; BR is involved in writing and supervision.

## Electronic supplementary material

Below is the link to the electronic supplementary material.

Online Resource 1: Figure S1 Development and evaluation of the fine-mapping population over several generations from the F3 to the F6 generation (PPTX 50 kb)

Online Resource 2: Table S2 List of molecular markers used in this project (XLSX 11 kb)

Online Resource 3: Table S3 List of the 70 genes within the 409-Kbp *Phg-2* region on chromosome 8. Annotations highlighted in red are candidate genes for resistance. Two closely related gene families depicted in yellow and blue. Genes that harbor missense mutations that segregate G10474 from the majority of other lines are shown in bold. (XLSX 13 kb)

Online Resource 4: Table S4 List of 117 SNPs in the 409-Kbp *Phg-2* region that differentiate G10474 against > 90% of further genotypes in a data set of whole-genome sequencing (WGS) data evaluated by Lobaton et al. (2018). Twenty SNPs that represent missense mutations are indicated in yellow; three of these that appear in LRR domain genes are shown in bold. (XLSX 40 kb)

Online Resource 5: Table S5 SNP identification for the design of new markers tagging the *Phg-2* resistance locus using WGS and GBS data on 600+ breeding lines, varieties and landraces. Consecutive filters are applied to select the best SNPs for marker design. A 3158 SNPs and INDEL polymorphisms identified between position 61,730,261 and 62,139,256 on 8 in the panel of 600+ lines. B 788 SNPs and INDELs that have genotyping calls for the three lines defined as source genotypes (MAB_348, MAB_349, G10474_wgs) C 576 polymorphisms are in the region where MAB 348/349 and G10474 seem to have the same haplotype, for 572 of those MAB 348/349 and G10474 have the same allele, 532 of those are biallelic. D 14 markers have a source allele frequency in the main population below 10%; this is essentially Table 4. (XLSX 8750 kb)
