# Supplementary figures and images for: Fine-mapping of angular leaf spot resistance gene Phg-2 in common bean and development of molecular breeding tools
Source: Theor Appl Genet. 2019 Apr 11;132(7):2003–16. doi: 10.1007/s00122-019-03334-z (PMC6588644; doi:10.1007/s00122-019-03334-z)

## Slide 1
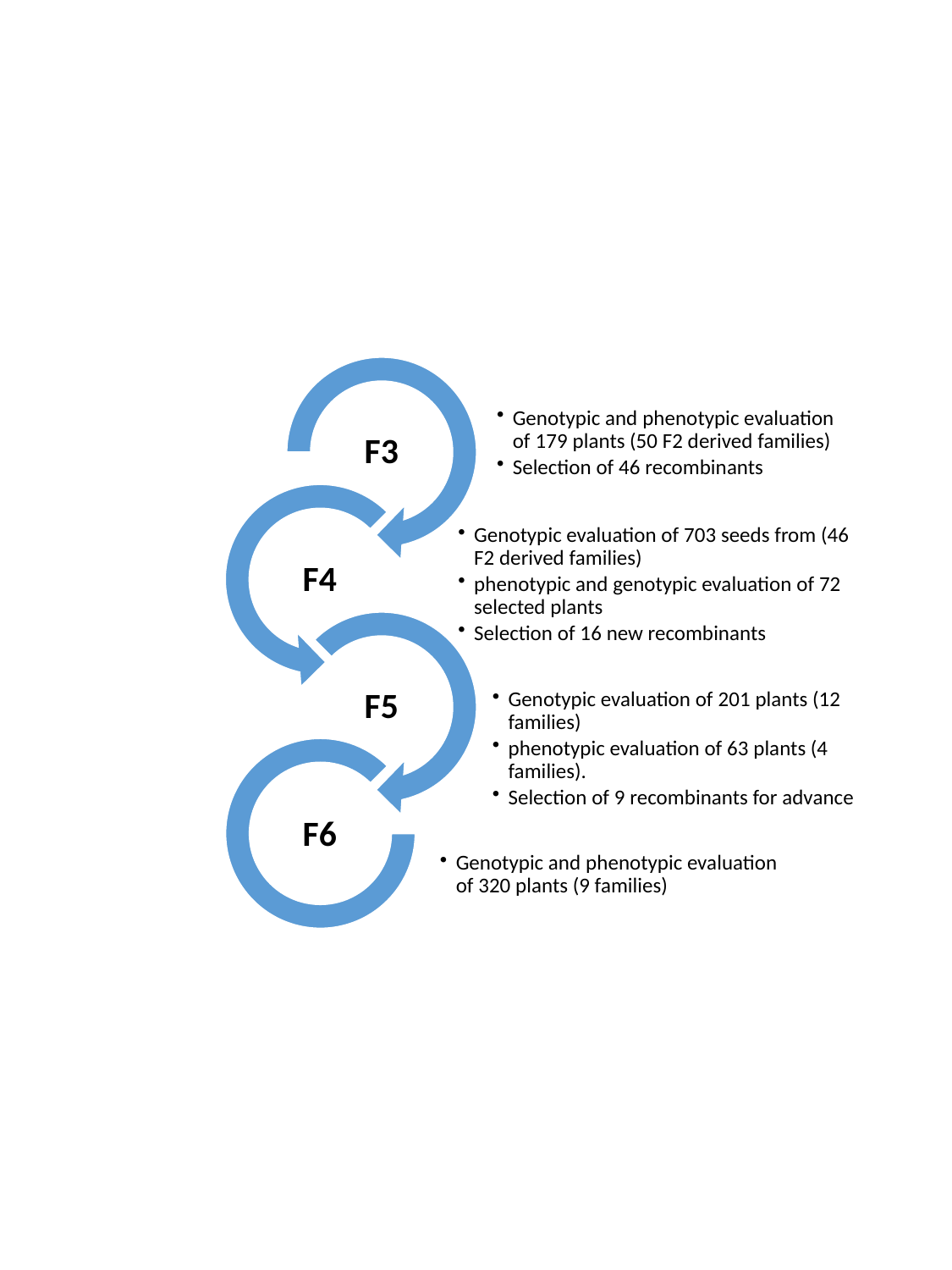

Supplement: Supplementary file 1 — Online Resource 1: Figure S1 Development and evaluation of the fine-mapping population over several generations from the F3 to the F6 generation (PPTX 50 kb) [file 122_2019_3334_MOESM1_ESM.pptx]
